# Extracellular Vesicles in Endometriosis: A Comprehensive Review of Biological Insights and Methodological Challenges

**DOI:** 10.3390/ijms27114666

**Published:** 2026-05-22

**Authors:** Aleksander Chodowiec, Magdalena Dec, Krzysztof Łuszczyński, Robert Zdanowski, Monika Szafarowska, Ludmiła Szewczak, Agnieszka Synowiec, Paweł Mitkowski, Paweł K. Włodarski, Anna Lutyńska, Aneta Ścieżyńska

**Affiliations:** 1 Laboratory of Molecular Oncology and Innovative Therapies, Military Institute of Medicine National Research Institute, 128 Szaserów Street, 04-141 Warsaw, Poland; achodowiec@wim.mil.pl (A.C.); mdec@wim.mil.pl (M.D.); kluszczynski@wim.mil.pl (K.Ł.); rzdanowski@wim.mil.pl (R.Z.); lszewczak@wim.mil.pl (L.S.); asynowiec@wim.mil.pl (A.S.); pmitkowski@wim.mil.pl (P.M.); alutynska@wim.mil.pl (A.L.); 2 Department of Histology and Embryology, Medical University of Warsaw, 02-004 Warsaw, Poland; pawel.wlodarski@wum.edu.pl; 3 Department of Gynecology and Oncological Gynecology, Military Institute of Medicine National Research Institute, 128 Szaserów Street, 04-141 Warsaw, Poland; mszafarowska@wim.mil.pl

**Keywords:** endometriosis, extracellular vesicles, microRNAs, biomarkers, EV isolation, MISEV guidelines

## Abstract

Endometriosis is a complex disorder associated with dysregulated immune, hormonal, and microenvironmental signaling. Extracellular vesicles (EVs) are important mediators of intercellular communication and may contribute to disease pathogenesis, biomarker discovery, and therapeutic targeting. Here, we systematically reviewed the literature on EVs in endometriosis, focusing on EV classification, isolation and characterization methods, and the functional relevance of EV-associated cargo. A total of 50 original studies were included and evaluated in the context of current International Society for Extracellular Vesicles (ISEV) recommendations. Our analysis revealed marked heterogeneity in EV nomenclature, biological sources, and methodological approaches. Although most studies used standard EV markers, the assessment of sample purity and inclusion of negative controls was inconsistent. Further studies using standardized workflows and well-characterized cohorts are needed to clarify their biological and clinical significance.

## 1. Introduction

Endometriosis is a highly prevalent chronic gynaecological disorder that is increasingly recognized as a condition involving dysregulated interactions between the nervous and immune systems [1]. It is defined by the presence of endometrial-like tissue outside the uterine cavity, most frequently affecting pelvic structures such as the ovaries, fallopian tubes, peritoneum, and bowel, with less common involvement of the urinary tract [2]. The disease exhibits marked heterogeneity in both extent and severity, ranging from superficial peritoneal implants to deep infiltrating endometriosis characterized by an invasion of pelvic organs, and, in rare cases, dissemination to extra-pelvic sites, including cutaneous and thoracic locations [2]. Importantly, endometriotic lesions may remain clinically silent and are identified in up to 50% of women undergoing infertility evaluation [1]. Endometriosis is often classified into three types based on lesion site and histological features: superficial peritoneal disease, ovarian endometriomas, and deep endometriosis. Superficial peritoneal lesions are usually limited to the peritoneal surface of pelvic tissues and are accompanied by minimal or no clinical symptoms. Ovarian endometriomas are cystic ovarian lesions with altered haemorrhagic content that are usually associated with reduced fertility and an increased risk of ovarian cancer. Deep endometriosis is distinguished by lesions that penetrate more than 5 mm into the peritoneal surface, frequently affecting visceral organs and compromising normal pelvic structure [2].

Large-scale population analyses indicate that endometriosis constitutes a substantial global health burden, contributing significantly to years lived with disability worldwide. Beyond its impact on reproductive health, epidemiological studies have consistently associated endometriosis with an increased risk of systemic comorbidities, including ovarian and breast cancer, melanoma, asthma, rheumatoid arthritis, and cardiovascular disease [1]. Population-based data further suggest that the true prevalence of endometriosis is likely underestimated, as community studies indicate that when both diagnosed and undiagnosed cases are considered, the condition may affect more than one in ten women [3]. Consequently, the chronic and multisystem nature of endometriosis substantially compromises quality of life in affected individuals.

Endometriosis is a complex, multifactorial disease whose pathogenesis remains incompletely understood. Multiple, non-mutually exclusive theories have been proposed to explain its origin, including retrograde menstruation, coelomic metaplasia, immune dysregulation, stem cell recruitment, and embryonic developmental remnants [2]. While retrograde menstruation may contribute to the establishment of ectopic endometrial lesions, it is insufficient to explain all disease phenotypes, particularly deep infiltrating and extraperitoneal endometriosis [2]. Accordingly, contemporary concepts of endometriosis emphasize the convergence of several biological processes rather than a single initiating event, reflecting the clinical and molecular heterogeneity of the disease.

Current evidence supports a model in which hormonal imbalance, altered immune responses, and epigenetic and microenvironmental factors collectively enable the survival, implantation, and progression of ectopic endometrial tissue [2]. Such aberrant immune responses are thought to arise from a proinflammatory cytokine milieu that promotes ectopic cell persistence. Moreover, for ectopic endometrial tissue to survive and expand, it must acquire enhanced proliferative capacity and resistance to apoptosis. Therefore, effective cell–cell communication is considered a fundamental mechanism regulating key physiological processes such as cell proliferation, apoptosis, development, and differentiation [4]. Accumulating evidence suggests that extracellular vesicles (EVs) may represent an important mode of intercellular crosstalk underlying these processes.

EVs are membrane-bound nanoparticles released by virtually all cell types, carrying a diverse cargo of proteins, lipids, metabolites, and nucleic acids enclosed within a lipid bilayer. Lacking a nucleus and replicative capacity, EVs function as key mediators of intercellular communication by transferring biologically active molecules between cells [5].

According to the recommendations of the International Society for Extracellular Vesicles (ISEV), EVs are currently classified based on their physical characteristics into two major categories: large EVs (lEVs; >200 nm in diameter) and small EVs (sEVs; <200 nm in diameter), rather than by their presumed biogenesis pathways. This standardized framework was introduced to address substantial heterogeneity and inconsistencies in EV nomenclature and experimental approaches across the literature [5].

EVs are involved in a wide range of physiological and pathological processes, including disease progression, biomarker discovery, and therapeutic targeting. In the context of endometriosis, EVs have emerged as potential regulators of immune responses, cellular proliferation, angiogenesis, and microenvironmental remodeling. However, studies investigating EVs in endometriosis remain highly heterogeneous, frequently employing inconsistent isolation strategies, overlapping terminology, and variable characterization criteria, which complicates data interpretation and cross-study comparison.

Given the methodological and conceptual variability, EV research in endometriosis requires a structured and guideline-compliant approach. In this study, we intend to organize existing data in line with current ISEV standards, resulting in an organized and repeatable framework for exploring EV-mediated processes in endometriosis.

## 2. Analysis of Current EV Studies in Endometriosis

*Extracellular Vesicle Isolation and Characterization in Endometriosis Research*

The publications presented in Table 1 analyzed extracellular vesicles derived from a variety of biological sources. Circulating biofluids, particularly blood plasma and serum, were the most commonly studied materials, accounting for the majority of samples. EVs were also isolated from peritoneal fluid, menstrual blood, and menstrual blood–derived stem cells, whereas uterine fluid, vaginal discharge, follicular fluid, and fallopian tube fluid were examined less frequently. In addition, several studies investigated EVs derived from primary endometrial stromal cells (ESCs), including both eutopic and ectopic ESC cultures.

The most commonly used approach for EV isolation was the ultracentrifugation-based method, including differential ultracentrifugation as well as ultracentrifugation combined with additional purification steps, such as iodixanol or sucrose density gradients. In several studies, size-exclusion chromatography (SEC) was applied, either alone or in combination with centrifugation-based procedures. Additionally, a substantial proportion of studies employed commercial precipitation or affinity-based kits, including ExoQuick, ExoQuick-TC, miRCURY Exosome Isolation kits, Exosupur purification kits, Total Exosome Isolation reagents, and other proprietary EV isolation systems. In some cases, EVs were isolated using sequential centrifugation protocols or combinations of centrifugation and commercial reagents (Table 1).

EVs were most frequently characterized using transmission electron microscopy (TEM), nanoparticle tracking analysis (NTA), and Western blotting (WB). Additional characterization approaches, including flow cytometry (FC) and dynamic light scattering (DLS), were reported in several studies, although a complete set of EV characterization methods was not provided in all cases (Table 1). The markers used for EV characterization included canonical EV markers such as CD9, CD63, CD81, HSP70, and TSG101, with CD63 and CD9 being the most frequently reported. In addition, several studies analyzed other EV-associated proteins, including Flotillin-1, Flotillin-2, Alix, Syntenin, Annexin V, Annexin A2, and the platelet marker CD61. The negative control marker calnexin was reported only in a limited number of studies.

Table 2 outlines the various types of EV-related studies included in the analysis. Across the analyzed papers, the most prevalent EV functional analyses were EV cargo profiling and gene expression analyses, while EV uptake assays and other functional assays, such as migration, proliferation, or co-culture experiments, were reported less frequently.

The majority of studies (19 out of 50) aimed to identify possible biomarkers. A large number of these studies focused on EV-associated non-coding RNAs, including microRNAs (miRNAs) and long non-coding RNAs (lncRNAs), in various tissues and body fluids. Several studies identified protein-based biomarkers derived from proteome analysis of EV cargo (Table 2).

A second significant group of studies (10 out of 50) examined EV-mediated immune-related modulation, including immune cell responses to EVs. Several studies in this category have demonstrated that EV cargo modulates macrophage phenotypes. Adding to the above, several papers (7 out of 50) investigated the impact of EVs on cellular behavior, such as proliferation, migration, and invasion. Some investigations found pro-angiogenic signaling, whereas others discovered fibrosis-related signaling associated with EV-derived molecules. Several articles (4 out of 50) found changed EV molecular profiles in endometriosis patients, based on EV cargo comparisons between patient and control groups. A subset of studies looked into EV-associated characteristics that influence reproductive outcomes, such as their effects on fertility-related processes.

## 3. Current Evidence and Methodological Challenges

### 3.1. Extracellular Vesicle Classification and Characterization in Endometriosis Research

**Figure 1:**
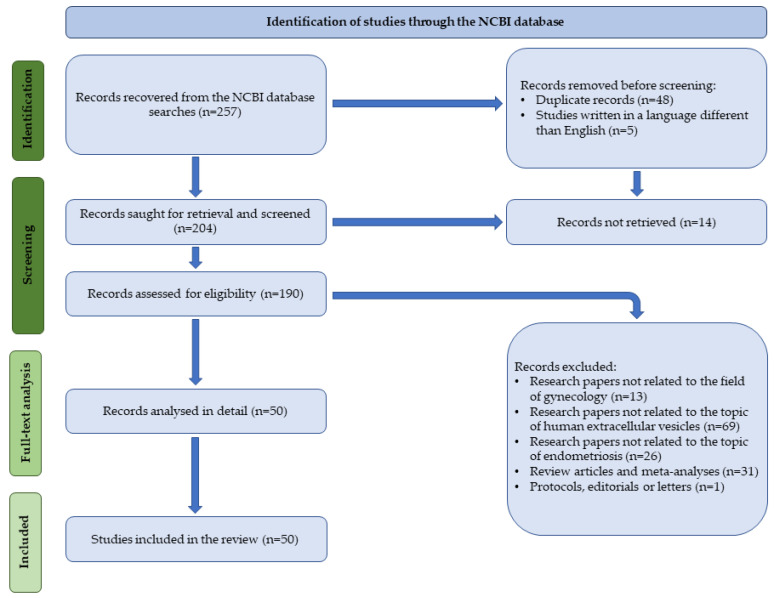
Flowchart illustrating the selection process of the analyzed articles.

Extracellular vesicles have emerged as important mediators in the pathogenesis and progression of a wide range of disorders, including cardiovascular diseases, neurological conditions, and cancer [56]. By contrast, in endometriosis research, only 50 identified studies met the criteria for detailed analysis, underscoring the still limited evidence base and the need for further investigation into the role of EVs in this disease (Figure 1, Table 1).

Extracellular vesicles are heterogeneous lipid bilayer-enclosed particles released by cells and can be classified according to their biogenesis, conceptual categories, or size [57]. The three classical EV subtypes are exosomes, microvesicles, and apoptotic bodies, distinguished mainly by their origin [57]. Exosomes arise from the endosomal system as intraluminal vesicles within multivesicular bodies and are released after fusion with the plasma membrane [56]. Their biogenesis involves Endosomal Sorting Complex Required for Transport (ESCRT) complexes and associated proteins such as ALIX and TSG101, although ESCRT-independent pathways have also been described [58]. Microvesicles are generated by outward budding of the plasma membrane, whereas apoptotic bodies are formed during programmed cell death [56,57].

However, according to the International Society for Extracellular Vesicles (ISEV), terms such as “exosomes” or “microvesicles” should be used only when a specific biogenesis pathway has been demonstrated [59]. Without analysis of markers specific to a given biogenesis pathway, ISEV recommends avoiding the use of such terms, as they imply a defined origin that is difficult to establish in isolated EV populations. Instead, EV subtypes should be described using operational terms referring to their physical characteristics, such as small EVs (sEVs) and medium/large EVs (m/lEVs), with defined size ranges (e.g., <200 nm or >200 nm), density (low, medium, high), or expression of specific surface molecules [59]. EV subtypes may also be classified based on their biochemical composition, for example, by the presence of markers such as CD63 [59]. Our analysis indicates that EV classification in endometriosis research remains only partially standardized (Table 1). Most studies referred to vesicles using operational terms such as small EVs (sEVs), whereas only a few distinguished between both small and large EV fractions, and a substantial proportion did not specify the EV subtype at all. This heterogeneity in terminology complicates comparisons between studies and may contribute to inconsistencies in reported findings.

The MISEV guidelines state that markers from five functional categories should be included in the characterization of EV proteins: (1) transmembrane or GPI-anchored proteins associated with the plasma membrane and/or endosomes, (2) cytosolic proteins recovered in EVs, (3) major components of non-EV co-isolated structures, (4) proteins associated with intracellular compartments other than the plasma membrane or endosomes and (5) secreted proteins recovered with EV preparations [59]. Additionally, to evaluate sample purity and exclude contamination from non-EV structures, EV analysis should include negative markers in addition to positive ones [59]. Our analysis revealed that most studies assessed proteins from categories 1 and 2, most commonly the traditional EV-associated markers CD63, CD9, CD81, TSG101, and HSP70, in accordance with these guidelines (Table 1). On the other hand, only a small proportion of studies included negative markers to exclude contamination from non-EV structures. Overall, these results indicate that while the majority of studies attempted to verify the vesicular nature of the analyzed preparations, the assessment of EV purity was less consistently addressed. These findings underscore the need for more consistent and transparent reporting in line with current ISEV recommendations. This issue is relevant for all research studies, not only in endometriosis research, as different EV fractions may be differentially enriched in regulatory RNAs, proteins, or other bioactive molecules. Consequently, variability in EV isolation and characterization strategies may have direct implications for study design, biomarker discovery, and the interpretation of downstream molecular analyses.

### 3.2. Methodological Variability in EV Isolation and Characterization

Methodological variability represents a major source of inconsistency in studies investigating EV-associated miRNAs. Different studies employed a wide range of EV isolation approaches, including differential ultracentrifugation, density gradient-based methods, size exclusion chromatography, fluid flow-based separation, ion exchange chromatography, and commercially available precipitation or affinity-based kits (Table 2). Although these methods are broadly accepted by MISEV, they differ substantially in yield, purity, and the spectrum of vesicles recovered, which can directly influence downstream miRNA profiling.

MISEV guidelines further emphasize the importance of rigorous EV characterization following isolation. This includes quantification of particle number using techniques such as nanoparticle tracking analysis (NTA), dynamic light scattering (DLS), flow cytometry, or microscopy-based approaches, as well as assessment of particle size distribution. In addition, evaluation of EV morphology using transmission electron microscopy (TEM), cryo-EM, scanning probe microscopy, or atomic force microscopy is recommended. Protein-based validation, including Western blot analysis with both positive and negative markers, as well as mass spectrometry and flow cytometry, is also advised to confirm EV identity and assess sample purity. Despite these recommendations, significant variability in isolation and characterization strategies persists. Importantly, MISEV2023 highlights that commercially available isolation kits should be used with caution, as they may introduce contaminants such as polymer-based reagents. Moreover, co-isolated molecules (including proteins, nucleic acids, lipids, and sugars) may form a dynamic EV “corona”, potentially influencing both biomarker profiles and functional readouts, and may be differentially retained or removed depending on the isolation method applied. Experimental evidence further underscores the impact of methodological differences. Morozumi et al. demonstrated that the choice of isolation method significantly affects EV purity, yield, and RNA content, indicating a fundamental trade-off between recovery and sample quality [60]. Similarly, Brennan et al. showed that serum-derived EV preparations vary markedly depending on the isolation technique, with high-yield methods such as precipitation and SEC associated with increased co-isolation of proteins and lipoproteins, whereas ultracentrifugation and density gradient approaches improved purity at the expense of particle recovery [61]. Consistently, Patil and Zhang reported that high-yield isolation strategies produced broader and less selective EV populations, while more selective approaches yielded fewer particles but higher purity and improved proteomic resolution [62]. Taken together, these findings indicate that no single isolation method is optimal for all applications, and that methodological choices can substantially influence EV composition and downstream analyses. This variability represents a critical challenge for cross-study comparisons and highlights the need for standardized protocols and careful interpretation of EV-associated miRNA data.

### 3.3. EV-Associated microRNAs in Endometriosis

Extracellular vesicles transport a wide range of molecules, including proteins, lipids, and nucleic acids such as miRNA, mRNA, and DNA, which can be delivered from donor to recipient cells, facilitating intercellular communication [56]. In the analysis performed here, the majority of studies focused on the miRNA cargo of EVs (Table 2). Indeed, miRNAs are involved in a wide range of cellular processes, including immune responses and antigen presentation, regulation of cellular metabolism, maintenance of homeostasis, and intercellular communication. Interestingly, there was only limited overlap between the EV-associated miRNAs identified across the analyzed studies. With the exception of miR-22-3p, which was reported in both biomarker and functional studies, most miRNAs were described in a single study (Table 2).

Several studies examined EV-associated miRNAs differentially expressed in patients with endometriosis compared to controls as potential biomarkers in bodily fluids (Table 2). Among these, miR-26b-5p, miR-215-5p, and miR-6795-3p were significantly altered and associated with disease severity [13]. Additionally, increased levels of miR-22-3p and miR-320a were observed in serum from patients with endometriosis [17]. The combined analysis of miR-22-3p and miR-320a yielded an area under the curve (AUC) of 0.883 (95% CI: 0.78–0.98; *p* < 0.01), indicating potential diagnostic value [17]. Another miRNA detected in serum, miR-214-3p, has been proposed as a potential biomarker, and functional analyses have demonstrated its role in inhibiting fibrosis in endometriosis through targeting connective tissue growth factor (CCN2) [22].

EV-associated miRNAs have also been identified in other biological fluids (Table 2). In menstrual blood-derived extracellular vesicles, miR-4443 levels were elevated in patients with endometriosis and positively correlated with dysmenorrhea (r = 0.42; *p* < 0.01) and dyspareunia (r = 0.64; *p* < 0.0001) [24]. The diagnostic performance of miR-4443 showed an AUC of 0.741 (95% CI: 0.624–0.858; *p* < 0.05), which increased to 0.929 (95% CI: 0.880–0.978; *p* < 0.05) when combined with dysmenorrhea [24]. Differential expression of EV-associated miRNAs was also observed in uterine luminal fluid, where miR-145-5p was significantly enriched in patients with endometriosis compared to controls [34]. Similarly, increased levels of miR-202-3p and miR-202-5p were detected in extracellular vesicles isolated from vaginal discharge [37].

Expression analyses performed in eutopic endometrium from patients with endometriosis revealed multiple dysregulated miRNAs, including upregulation of miR-210-3p, which was also enriched in extracellular vesicles isolated from uterine fluid [33]. Functional studies have demonstrated that miR-210-3p may contribute to immune evasion through modulation of the JNK signaling pathway [32] (Table 2).

EV-associated miRNAs have also been implicated in immune-related processes. Analyses of peritoneal fluid samples identified differentially expressed exosomal miRNAs in patients with endometriosis, accompanied by increased levels of inflammatory cytokines and altered immune cell populations, including myeloid-derived suppressor cells and regulatory T cells [29]. Additionally, several studies have demonstrated that EV-associated miRNAs participate in macrophage polarization. Exosomes derived from the uterine cavity increased the expression of miR-210-3p and induced M2 macrophage polarization through the regulation of ATP5D [33]. Similarly, exosomes released from ectopic endometrial stromal cells promoted M2 macrophage polarization by delivering miR-146a-5p via TRAF6 signaling [47]. In addition, exosomal miR-301a-3p derived from endometriosis tissues promoted macrophage polarization through regulation of the PTEN–PI3K signaling axis [20]. Exosomal miR-22-3p released from peritoneal macrophages was transferred to ectopic endometrial stromal cells and promoted their proliferation, migration, and invasion via the SIRT1/NF-κB pathway [31] (Table 2).

EV-associated miRNAs have also been linked to processes involved in lesion progression, including angiogenesis (Table 2). Exosomes derived from ectopic endometrial stromal cells were found to promote endothelial cell proliferation, migration, and tube formation, effects associated with miR-21-5p targeting TIMP3 [48]. EV-associated miRNAs have also been associated with fibrosis and extracellular matrix remodeling. Downregulation of miR-214-3p was accompanied by increased expression of its target Connective Tissue Growth Factor (CCN2). Functional analyses confirmed that exosomal miR-214-3p inhibited fibrosis through regulation of CCN2 [22]. In addition, extracellular vesicles derived from ectopic stromal cells contained elevated levels of miR-25-3p, which promoted collagen I deposition and was associated with decreased Phosphatase and Tensin homolog (PTEN) expression and increased p-Akt signaling in recipient cells [43].

Finally, EV-associated miRNAs may influence reproductive processes (Table 2). In uterine luminal fluid, extracellular vesicles enriched in miR-145-5p impaired blastocyst development and were associated with altered expression of components of the NOTCH signaling pathway [34]. Furthermore, EV-mediated transfer of miR-25-3p may affect decidualization processes, thereby indirectly influencing embryo implantation [43].

## 4. Outlook and Conclusions

Several limitations emerge from the current body of literature on EV-associated miRNAs in endometriosis (Table 1 and Table 2). First, many studies employ small patient cohorts, which restricts statistical power and affects the general applicability of the findings. This is especially important in the context of biomarker discovery, when thorough validation across larger cohorts is required. Second, the biological material examined is relatively heterogeneous. EV-associated miRNAs have been studied in a variety of sample types, including serum, plasma, menstrual blood, peritoneal fluid, uterine luminal fluid, endometrial tissue, and cell cultures. While this diversity reflects the widespread interest in EV biology, it makes direct comparisons between research difficult and may contribute to the minimal overlap of discovered miRNA signatures. Differences in EV characterization methodologies further reduce comparability. Not all research followed standard protocols for EV validation, and the level of marker-based characterization and contamination control differed greatly between investigations. Finally, the utilization of different experimental models, such as primary tissues, biofluids, and in vitro cell systems, complicates interpretation. While each model offers useful insights, differences in cellular origin and microenvironment may have a considerable impact on EV cargo composition. Overall, these observations highlight the need for further studies to validate EV-associated miRNAs in endometriosis and to define their diagnostic and mechanistic significance.

Despite these limitations, the available data indicate that EVs are likely to be actively involved in the endometriotic microenvironment rather than merely reflecting ongoing cellular activity. Their association with immune regulation, macrophage polarization, angiogenesis, fibrosis, and fertility-related processes suggests that they may contribute to several key aspects of disease development and progression. This makes EVs relevant not only as a potential source of biomarkers but also as a useful framework for studying disease mechanisms and identifying new therapeutic directions. At the same time, the field remains methodologically challenging. Further progress in the field will require larger and well-characterized cohorts, standardized EV isolation and characterization workflows, and rigorous validation of candidate EV-associated molecules and pathways. Such efforts will be essential to clarify the biological role of EVs in endometriosis, improve reproducibility across studies in line with current MISEV2023 recommendations, and determine their true diagnostic and therapeutic potential.

## 5. Methodology of the Literature Selection Process

Literature was retrieved from the National Center for Biotechnology Information (NCBI) database (https://www.ncbi.nlm.nih.gov/, accessed on 2 March 2026). The following search terms were used in various combinations with the Boolean operators “AND” and “OR”: “Exosomes”, “Human”, “Endometriosis”, “Menstrual Blood”, “Microvesicles”, and “Vesicles”.

A flowchart summarizing the literature selection process is presented in Figure 1. Initially, 257 articles in the NCBI database were found. Following removal of duplicates (*n* = 48) and papers written in languages other than English (*n* = 5), and records not retrieved (*n* = 14), 190 articles remained for screening. Title and abstract screening resulted in the further exclusion of 140 records, primarily due to lack of relevance to human extracellular vesicles (*n* = 69), gynecology and endometriosis (*n* = 39), or because the articles were review papers rather than original research (*n* = 32). Finally, 50 studies met the inclusion criteria and were incorporated into the analysis.

The included studies were systematically evaluated for the following parameters: study group (including the number of patients and healthy controls), source of extracellular vesicles (EVs), isolation and characterization techniques, and their compliance with the MISEV2023 guidelines [5]. Additionally, extracted data included subtype classification of EVs and applied analytical methods.

## Figures and Tables

**Table 1:** Methodological overview of extracellular vesicle isolation, characterization, and marker analysis in endometriosis research.

Ref.	Endo.	Cont.	EV Sample Source	EV Isolation Method	Additional Purification/ Reagent	Phenotype Characterization Techniques					Analyzed Markers					
						(T)EM	NTA	WB	FC	Other	CD9	CD63	CD81	HSP70	TSG101	Other
[6]	5	6	Blood plasma	Affinity-based isolation	exoRNeasy Serum/Plasma Midi Kit (Qiagen, Hilden, Germany)	+	+	+				+				
[7]	30	10	Blood plasma	Centrifugation	-				+							MMP9, VEGF
[8]	25 * (adenomyosis)	31	Blood plasma	Differential ultracentrifugation	Iodixanol gradient	+	+	+		LVSEM, LC-MS	+		+			Flotillin-2
[9]	12	12	Blood plasma	Ultracentrifugation	-		+	+			+					Alix, calnexin
[10]	45	15	Blood plasma	Ultracentrifugation	-	N/A	N/A	N/A	N/A	N/A	N/A	N/A	N/A	N/A	N/A	N/A
[11]	6	6	Blood plasma	Ultracentrifugation + Precipitation-based isolation	miRCURY Exosome Isolation Kit (Exiqon, Vedbæk, Denmark)	+		+				+				Calnexin
[12]	86	43	Blood plasma	N/A	-	N/A	N/A	N/A	N/A	N/A	N/A	N/A	N/A	N/A	N/A	N/A
[13]	32	24	Serum	Affinity-based isolation	Exosome Binding Enhancer (Wako, Tokyo, Japan)	+		+			+	+				
[14]	30	16	Serum	Polymer-based precipitation	ExoQuick Exosome Precipitation Solution (System Biosciences, Palo Alto, CA, USA)	+		+				+			+	
[15]	29	16	Serum	Polymer-based precipitation	ExoQuick Exosome Precipitation Solution (System Biosciences, USA)	+	+	+			+	+		+	+	
[16]	52	21	Serum	Polymer-based precipitation	ExoQuick Exosome Precipitation Solution (System Biosciences, USA)	+	+	+			+	+				Calnexin
[17]	25	25	Serum	Differential centrifugation		+	+	+			+	+				
[18]	7	6	Serum	Ultracentrifugation	Sucrose gradient	+	+		+			+	+			
[19]	14	34	Serum	Ultracentrifugation		+	+	+			+	+	+			
[20]	N/A	N/A	Serum	Ultracentrifugation		+	+	+				+			+	Calnexin
[21]	111	35	Serum	Size-exclusion chromatography (SEC)		+	+	+								VEGF-C
[22]	20	20	Serum	N/A												-
[23]	8	9	Menstrual blood	Differential centrifugation		+	+	+	+		+	+	+			
[24]	5	7	Menstrual blood	Differential centrifugation → ultracentrifugation		+	+	+			+		+			Flotillin-1
[25]	n/a	n/a	Menstrual blood stem cells	Polymer-based precipitation	EXOCIB Exosome Isolation Kit (Cib Biotech, Shiraz, Iran)	+			+	DLS	+	+	+			
[26]	5	10	Menstrual blood stem cells	Polymer-based precipitation	EXOCIB Exosome Isolation Kit (Cib Biotech, Iran)	+			+	DLS	+	+	+			
[27]	6	6	Peritoneal fluid	Polymer-based precipitation	Total Exosome Isolation Reagent (Invitrogen, Carlsbad, CA, USA)				+	AFM						CD61
[7]	26	11	Peritoneal fluid	Centrifugation					+							MMP9, VEGF
[28]	22	6	Peritoneal fluid	Centrifugation → SEC		+	+	+			+					Syntenin, Alix
[29]	54	13	Peritoneal fluid	Differential centrifugation		N/A	N/A	N/A	N/A	N/A	N/A	N/A	N/A	N/A	N/A	N/A
[24]	6	6	Peritoneal fluid	Differential centrifugation		+	+	+			+		+			Flotillin-1
[30]	50	50	Peritoneal fluid macrophage culture	Sequential centrifugation		+	+	+			+	+				
[11]	6	6	Peritoneal fluid	Ultracentrifugation + Precipitation-based isolation	miRCURY Exosome Isolation Kit (Exiqon, Denmark)	+		+				+				Calnexin
[10]	45	15	Peritoneal fluid	Ultracentrifugation		N/A	N/A	N/A	N/A	N/A	N/A	N/A	N/A	N/A	N/A	N/A
[31]	20	20	Macrophage from peritoneal fluid	Differential centrifugation		+	+	+			+	+				
[32]	22	25	Uterine fluid	Size-exclusion chromatography (SEC)	Exosupur Exosome Purification Kit (Echo Biotech, Beijing, China)	+	+	+				+		+	+	Calnexin
[33]	N/A	N/A	Uterine fluid	Size-exclusion chromatography (SEC)	Exosupur Exosome Purification Kit (Echo Biotech, China)	+	+	+				+		+	+	
[34]	30	30	Uterine fluid	Ultracentrifugation		+	+	+			+	+	+			Calnexin
[35]	10	10	Vaginal discharge	Polymer-based precipitation	ExoQuick Exosome Precipitation Solution (Cell Guidance Systems, Cambridge, UK)	+	+				N/A	N/A	N/A	N/A	N/A	N/A
[36]	26	25	Vaginal discharge	Differential centrifugation		+	+	+				+		+		Flotillin-1, Calnexin
[37]	11	11	Vaginal discharge	Differential ultracentrifugation		+	+	+				+		+		
[38]	14	13	Endometriomas	Polymer-based precipitation	ExoQuick Exosome Precipitation Solution (System Biosciences, USA)	+		+			+	+	+	+		
[8]	31, but adenomyosis	0	Adenomyotic tissue	Differential ultracentrifugation	iodixanol gradient	+	+	+		LVSEM, LC-MS	+		+			Flotillin-2
[36]	26	25	Endometrial ectopic tissue	Differential centrifugation		+	+	+				+		+		Flotillin-1, Calnexin
[20]	n/a	n/a	Ectopic endometrial tissue	Ultracentrifugation		+	+	+				+			+	Calnexin
[11]	6	6	Endometriotic tissue	Ultracentrifugation + Precipitation-based isolation	miRCURY Exosome Isolation Kit (Exiqon, Denmark)	+		+				+				Calnexin
[39]	5	5	Eutopic ESC	Ultracentrifugation + Commercial kit	Exosome Isolation Kit (Invitrogen)	+	+				N/A	N/A	N/A	N/A	N/A	N/A
[21]	111	35	Eutopic ESC	Polymer-based precipitation	ExoQuick Exosome Precipitation Solution (System Biosciences, USA)	+	+	+								VEGF-C
[40]	3	3	Eutopic ESC	Polymer-based precipitation	ExoQuick Exosome Precipitation Solution (System Biosciences, USA)	+			+			+	+			
[41]	10	10	Eutopic ESC	Polymer-based precipitation	ExoQuick Exosome Precipitation Solution (System Biosciences, USA)	N/A	N/A	N/A	N/A	N/A	N/A	N/A	N/A	N/A	N/A	N/A
[42]	50	50	Eutopic ESC	Sequential centrifugation		+	+	+			+	+				
[43]	6	6	Eutopic ESC	Differential centrifugation		+	+	+				+			+	Alix, Calnexin
[37]	6	5	Eutopic ESC	Differential ultracentrifugation		+	+	+				+		+		
[44]	3	n/a	Eutopic ESC	Differential centrifugation		+	+	+				+	+	+	+	
[45]	20	20	Eutopic ESC	Ultracentrifugation		+	+	+				+			+	Alix, Calnexin, GM130
[11]	6	6	Eutopic ESC	Ultracentrifugation + Precipitation-based isolation	miRCURY Exosome Isolation Kit (Exiqon, Denmark)	+		+				+				Calnexin
[46]	30	20	Ectopic ESC	Polymer-based precipitation	Exo Extraction Kit (ExoQuick; System Biosciences, USA)	+	+	+			+	+	+		+	
[47]	n/a	n/a	Ectopic ESC	Polymer-based precipitation	Exo Extraction Kit (ExoQuick; System Biosciences, USA)	+		+					+		+	
[46]	30	20	Eutopic ESC	Polymer-based precipitation	Exo Extraction Kit (ExoQuick; System Biosciences, USA)	+	+	+			+	+	+		+	
[47]	n/a	n/a	Eutopic ESC	Polymer-based precipitation	Exo Extraction Kit (ExoQuick; System Biosciences, USA)	+		+					+		+	
[15]	10	10	Ectopic ESC	Polymer-based precipitation	Total Exosome Isolation Reagent (Thermo Fisher Scientific, Waltham, MA, USA)	+	+	+			+	+		+	+	
[39]	5	5	Ectopic ESC	Ultracentrifugation + Polymer-based precipitation	Total Exosome Isolation Reagent (Thermo Fisher Scientific, USA)	+	+				N/A	N/A	N/A	N/A	N/A	N/A
[16]	2	0	Ectopic ESC	Polymer-based precipitation	ExoQuick Exosome Precipitation Solution (System Biosciences, USA)	+	+	+			+	+				Calnexin
[21]	111	35	Ectopic ESC	Polymer-based precipitation	ExoQuick Exosome Precipitation Solution (System Biosciences, USA)	+	+	+								VEGF-C
[22]	20	20	Ectopic ESC	Polymer-based precipitation	ExoQuick Exosome Precipitation Solution (System Biosciences, USA)	+		+			+	+				
[14]	30	16	Ectopic ESC	Polymer-based precipitation + centrifugation	Total Exosome Isolation Reagent (Thermo Fisher Scientific, USA)	+		+				+			+	
[43]	6	6	Ectopic ESC	Differential centrifugation		+	+	+				+			+	Alix, Calnexin
[37]	6	5	Ectopic ESC	Differential ultracentrifugation		+	+	+				+		+		
[45]	20	20	Ectopic ESC	Ultracentrifugation		+	+	+				+			+	Alix, Calnexin, GM130
[44]	3	n/a	Ectopic ESC	Differential centrifugation		+	+	+				+	+	+	+	
[48]	50	50	Ectopic ESC	Ultracentrifugation		+	+	+	+			+	+			Calnexin
[11]	6	6	Ectopic ESC	Ultracentrifugation + Precipitation-based isolation	miRCURY Exosome Isolation Kit (Exiqon, Denmark)	+		+				+				Calnexin
[18]	5	0	Other: cells isolated from endometriotic tissue	Ultracentrifugation	Sucrose gradient	+	+		+			+	+			CA125
[49]	13	13	Other: cells isolated from ovarian endometrioma and endometriotic tissue	Polymer-based precipitation	ExoQuick Exosome Precipitation Solution (System Biosciences, USA)	+	+	+			N/A	N/A	N/A	N/A	N/A	N/A
[50]	8	0	Other: ectopic endometrioma wall explant culture	Differential centrifugation		+	+		+		+		+			
[51]	15	8	Other: hUC-MSCs	Ultracentrifugation	Exosome Extraction Kit (Bioruo, Beijing, China)	+	+	+			+	+	+	+		
[52]	n/a	n/a	Other: hUC-MSCs	Ultracentrifugation		+	+		+			+	+			
[53]	5	6	Other: hUC-MSCs	Principle not reported	Exosome Extraction Kit (Bioruo, China)	+					N/A	N/A	N/A	N/A	N/A	N/A
[27]	6	6	Other: Follicular fluid	Centrifugation	Total Exosome Isolation Reagent (Thermo Fisher Scientific, USA)				+	AFM						CD61
[54]	10 (*) endometrioma	10	Other: Follicular fluid	Ultracentrifugation		+	+	+			+	+				
[55]	4	5	Other: Fallopian tube fluid	Ultracentrifugation		+	+	+			+				+	Flotillin-1
[24]	4	5	Other: Fallopian tube fluid	Differential centrifugation		+	+	+			+		+			Flotillin-1

**Abbreviations: Endo.**, number of samples obtained from patients with endometriosis; **Cont.**, number of samples obtained from healthy controls; **EV**, extracellular vesicle; **(T)EM**, (transmission) electron microscopy; **NTA**, nanoparticle tracking analysis; **WB**, Western blot; **FC**, flow cytometry; **LVSEM**, low-voltage scanning electron microscopy; **LC-MS**, liquid chromatography–mass spectrometry; **DLS**, dynamic light scattering; **AFM**, atomic force microscopy; **SEC**, size-exclusion chromatography; **ESC**, endometrial stromal cells; **hUC-MSCs**, human umbilical cord-derived mesenchymal stem cells; **VEGF**, vascular endothelial growth factor; **VEGF-C**, vascular endothelial growth factor C. **N/A** information not provided; * samples with adenomyosis were analyzed.

**Table 2:** Functional analyses and reported outcomes of extracellular vesicle studies in endometriosis.

Publication	EV Sample Source	Implemented EV Analyses:				Conclusions
		EV Cargo Analysis *	Gene Expression	EV Uptake	Further Analyses	
[8]	Blood plasma, Adenomyotic tissue	Proteomics				Identification of potential biomarkers, Influence on cell invasion
[6]	Blood plasma	lncRNA				Identification of potential biomarker: EV-associated lncRNAs
[21]	Serum, Eutopic, Ectopic ESC		(Gene knockout studies)	+	Transwell migration, cell proliferation, Western blot	Identification of potential biomarker: VEGF-C; Pro-angiogenic signalling
[19]	Serum	miRNA				Identification of potential biomarker: EV miRNAs associated with reproductive outcomes
[13]	Serum	miRNA				Identification of potential biomarker: EV miRNAs
[17]	Serum	miRNA				Identification of potential biomarker: miR-22-3p, miR-320a
[14]	Serum, Ectopic ESC		+	+	Tube formation, wound healing	Identification of potential biomarker: antisense hypoxia-inducing factor (aHIF); Pro-angiogenic signalling
[15]	Serum, Ectopic ESC		+	+	Transwell migration; Wound healing	Identification of potential biomarker: lncRNA TC0101441; Involvement in transport of metastasis factors
[16]	Serum, Ectopic ESC		+	+		Identification of potential biomarker: EV-LGMNP1; Macrophage phenotype modulation: LGMNP1
[22]	Serum, Ectopic ESC				Effects of EV-derived miRNA	Identification of potential biomarker: miR-214-3p; Fibrosis-related signalling
[23]	Menstrual blood	Proteomics			EV co-culture with mesothelial cells	Identification of potential biomarker: EV proteins’ role in endometriosis lesion establishment
[24]	Menstrual blood, Peritoneal fluid, Fallopian tube fluid	miRNA		+		Identification of potential biomarker: miR-4443, contributing to endometriosis pathogenesis
[27]	Peritoneal fluid				Network analysis: proteomic STRING analysis	Identification of potential biomarker: platelet-derived EVs
[28]	Peritoneal fluid	Proteomics				Identification of potential biomarker: PRDX1, ANXA2, ITIH4
[54]	Follicular fluid		+			Identification of potential biomarker, Altered EV molecular profile
[36]	Endometriotic tissue, Vaginal discharge		+		Pathway analysis: proteomics (KEGG)	Identification of potential biomarker: tRF-Leu-AAG-001 (promoting inflammation and angiogenesis)
[37]	Vaginal discharge, Eutopic ESC, Ectopic ESC	miRNA				Identification of potential biomarker: miR-202-3p, miR-202-5p
[42]	Eutopic ESC		+	+	Transwell migration; Wound healing; Tube formation; Analysis of EV lncRNA effects	Identification of potential biomarker: EV lncRNA sponges up miR-761
[49]	Other: cells are isolated from endometriotic tissue and endometrioma				Network analysis: construction and topological analysis of exosomal RNA network	Identification of potential biomarker: circRNA–miRNA–mRNA network
[18]	Serum, Other: cells isolated from endometriotic tissue				EV surface analysis: IF, FC	EV-mediated immune modulation (NK activity impairment)
[30]	Peritoneal fluid macrophage culture		+	+	Transwell migration; wound healing, Dual reporter gene assay	EV-mediated immune modulation: lncRNA CHL-AS1 (sponge for miR-610)
[29]	Peritoneal fluid	miRNA				EV-mediated immune modulation
[31]	Macrophage from peritoneal fluid		+	+	Transwell migration; Wound healing	EV-mediated immune modulation: miR-22-3p
[38]	Endometriomas				Ectonucleotidase activity	EV-mediated immune modulation
[9]	Blood plasma			+		Macrophage phenotype modulation
[33]	Uterine fluid	miRNA		+	Wound healing; Colony formation, Decidualization assay, Transwell migration	Macrophage phenotype modulation: miR-210-3p
[32]	Uterine fluid	miRNA	+	+	Transwell migration; EV co-culture with macrophages	Macrophage phenotype modulation:miR-210-3p
[47]	Ectopic ESC	miRNA			Co-culture with macrophages	Macrophage phenotype modulation: miR-146a-5p
[20]	Ectopic endometrial tissue, serum	miRNA		+		Macrophage phenotype modulation: miR-301a-3p
[7]	Blood plasma, Peritoneal fluid	+				Pro-angiogenic signalling
[39]	Eutopic ESC	miRNA			Tube formation	Pro-angiogenic signalling (miR-21)
[48]	Ectopic ESC	miRNA		+	Proliferation; wound healing; Tube formation; transwell migration	Pro-angiogenic, enhancing proliferation and migration signalling: miR-21-5p
[43]	Eutopic ESC, Ectopic ESC	miRNA		+	EV co-culture with ESCs	Fibrosis-related signalling: miR-25-3p
[45]	Eutopic ESC, Ectopic ESC		+		EV co-culture with ESCs	Fibrosis-related signalling: PKM2
[40]	Eutopic ESC	miRNA	+			Influence on fertility
[34]	Uterine fluid	miRNA				Influence on fertility: miR-145-5p (negative impact)
[35]	Vaginal discharge				EV co-culture with human sperm	Influence on fertility
[46]	Ectopic ESC, Eutopic ESC		+	+	Transwell migration	Influence on cell proliferation and migration: AFAP1-AS1
[44]	Ectopic ESC, Eutopic ESC	Proteomics		+	Transwell migration, Tube formation	Influence on cell proliferation and migration: Annexin A2
[51]	Other: hUC-MSCs	miRNA	+	+	Transwell migration; Wound healing	Influence on cell proliferation and migration: miR-100
[11]	Blood plasma, Peritoneal fluid, Ectopic ESC, Eutopic ESC, Endometriotic tissue	Proteomics, miRNA		+		Altered EV molecular profile in patients with endometriosis
[12]	Blood plasma				cMP-TF activity (ELISA)	Altered EV molecular profile and levels of EV in patients with endometriosis
[41]	Eutopic ESC	miRNA			Apoptosis; Transwell migration; Wound healing; Proliferation	Altered EV molecular profile, influence on cell proliferation
[50]	Ectopic endometrioma wall explant culture	miRNA				Altered EV molecular profile
[26]	Menstrual blood-derived stem cells		+		Apoptosis, Wound healing (scratch); IF; ELISA	Gene expression modulation of EVs, influence on cell proliferation and migration
[55]	Other: Fallopian tube fluid	miRNA	+			Gene expression modulation
[10]	Blood plasma, Peritoneal fluid		+			Metabolic pathway alterations
[25]	Menstrual blood-derived stem cells				NEMenSC-derived EV effects on E-MenSCs (apoptosis, inflammatory markers)	Potential therapeutic effect of EVs
[52]	Other: hUC-MSCs		+		Matrigel invasion, Western blot,	Potential therapeutic effect of hUC-MSC-derived EVs from healthy patients
[53]	Other: hUC-MSCs		+	+	Transwell migration; Wound healing; Western blot	Potential therapeutic effect

**Abbreviations: EV**, extracellular vesicle; **ESC**, endometrial stromal cells; **lncRNA**, long non-coding RNA; **IF**, immunofluorescence; **FC**, flow cytometry; **ELISA**, enzyme-linked immunosorbent assay; **hUC-MSCs**, human umbilical cord-derived mesenchymal stem cells; **NEMenSCs**, normal endometrial mesenchymal stem cells; **E-MenSCs**, endometriotic mesenchymal stem cells; **NK**, natural killer; **WB**, Western blot. * EV cargo analysis includes proteomic, miRNA, lncRNA, circRNA and other molecular profiling approaches.

## Data Availability

No new data were created or analyzed in this study. Data sharing is not applicable to this article.

## Abbreviations

The following abbreviations are used in this manuscript:

ISEV	International Society for Extracellular Vesicles
EV	Extracellular Vesicles
ESC	Endometrial Stem Cells
WB	Western Blot
NTA	Nanoparticle Tracking Analysis
FC	Flow Cytometry
TEM	Transmission Electron Microscopy
DLS	Dynamic Light Scattering
